# Barriers and opportunities in the translation of mobile phone and social media interventions between research and health promotion practice in Australia: a qualitative study of expert perspectives

**DOI:** 10.1186/s12961-018-0406-x

**Published:** 2019-01-10

**Authors:** Cassandra J. C. Wright, Joanna Schwarzman, Paul M. Dietze, Belinda Crockett, Megan S. C. Lim

**Affiliations:** 1 Centre for Population Health, Burnet Institute, 85 Commercial Rd, Melbourne, Victoria 3004 Australia; 2 Department of Epidemiology and Preventive Medicine, Monash University, 99 Commercial Rd, Melbourne, Victoria Australia; 3 Melbourne School of Population and Global Health, University of Melbourne, 235 Bouverie St, Carlton, Victoria Australia

**Keywords:** Australia, health promotion, knowledge translation, qualitative, social media, mobile phones

## Abstract

**Background:**

Newer technologies, such as smartphones and social networking sites, offer new opportunities for health promotion interventions. There is evidence to show that these technologies can be effectively and acceptably used for health promotion activities. However, most interventions produced in research do not end up benefitting non-research populations, while the majority of technology-facilitated interventions which are available outside of research settings are either undocumented or have limited or no evidence to support any benefit. We therefore aimed to explore the perspectives of researchers and health promotion experts on efforts to translate technology-facilitated prevention initiatives into practice, and the barriers to achieving translation.

**Methods:**

We utilised a qualitative study design, involving in-depth interviews with researchers experienced with technology-facilitated prevention interventions and prominent health promotion experts.

**Results:**

Some barriers mirror the findings of other studies into health promotion practice, which have found that competing priorities, resource limitations and organisational capacity are important in determining use of evidence in programme planning, engagement in translation and evaluation practice. We add to this literature by describing barriers that are more specifically related to technology-facilitated prevention, such as the pace of developments in technology, and how this clashes with the time taken to develop and ready evidence for translation.

**Conclusions:**

In order to maximise the vast potential of technology-facilitated prevention interventions to promote population health, it is essential that translation is at the forefront of consideration for both researchers and practitioners. We suggest actions that can be taken by both researchers and practitioners to improve translation of technology-facilitated prevention interventions, and also highlight how funding schemes can be modified to facilitate translation.

## Introduction

Newer technologies such as smartphones and social media offer innovative opportunities for reaching populations with health promotion interventions [1]. Evidence shows that these technologies can be effectively and acceptably used for health promotion activities, including provision of health information, delivery of behaviour change interventions, symptom monitoring for self-management of diseases, awareness-raising and advocacy strategies [1–4]. Popular domains of study include tobacco cessation [1, 5, 6], medication adherence [7], chronic disease self-management [1, 5, 7], brief interventions for substance use [8, 9], symptom monitoring and support for mental health conditions [10, 11], and education and motivational messaging for sexual health [12–14], physical activity [15–17] and diet [17–19]. We will refer to these types of activities as technology-facilitated prevention interventions throughout this paper.

One of the main benefits of using technology for prevention programmes is its wide reach, offering low-cost opportunities to connect with populations at a large scale and low participant burden due to the high level of integration of mobile phones and social media into people’s daily routines [20–22]. Functional benefits of mobile phones and social media include the potential for real-time monitoring and feedback of participant behaviours and health indicators [23], complex intervention tailoring [24], and modes of communication that can be highly interactive, visually appealing and engaging [20]. A further major benefit is that real-time usage data can be automatically generated within most technology platforms, which can be advantageous for evaluation purposes [20].

The potential for technology to be used for prevention gains has been the focus of much attention in both the research and practice sectors. Over the past decade, products and campaigns have emerged from the fields of medicine, allied health, health promotion, behavioural science, psychology, marketing, communications and education, each bringing different approaches and theories. While there is growing evidence to support the use of mobile phones and social media for reducing risk behaviours and improving health and wellbeing, there is a clear difference between the interventions developed and tested in research settings and interventions implemented by health promotion practitioners and others in the ‘real world’ [6, 25].

In the research sector, most of the technology-facilitated prevention work focuses on behaviour change interventions, and most studies are small-scale pilot interventions [26]. Studies frequently describe the potential for interventions to be scaled up (i.e. expanded under real-world conditions into practice to reach larger populations [27]), but evidence of larger studies and/or attempts to implement technology-facilitated interventions into community settings are far rarer [26]. This follows the major trends of intervention studies in general, which are most likely to exist in small, pilot trial form, and often not translated to reach non-research populations [27, 28]. Other critiques of technology-facilitated prevention research include inadequate description of implementation requirements (including cost and human resourcing), and lack of dissemination in plain language and outside of academic journals [29]. Some work has been done to update reporting requirements of eHealth and mHealth interventions [30, 31], but few journals publishing technology-facilitated prevention work require adherence to these criteria.

In the Australian context, health promotion or prevention activities that reach the public are implemented by a range of organisation types. These include government (at a local, state and national level), non-government (such as community health services) and not-for-profit organisations or charities focused on specific health issues (i.e. Diabetes Australia). At a national and state level, investment in prevention has been in flux for the past two decades and, as such, there have been few time-points at which funding has been considered to be relatively stable [32–34]. These organisations conduct a range of activities across the health promotion spectrum, from action focused at the policy and environment level through to initiatives focused at the individual level [34]. Australia’s health promotion sector has delivered world-leading evidence-based initiatives in particular domains such as tobacco and HIV-related policy and programmes [35]. However, there are many other areas in which knowledge translation has not occurred effectively, and we have failed to make meaningful progress; a prime example of this is Indigenous health and wellbeing [34].

There are relatively few Australian publications (in either grey or academic literature) that describe technology-facilitated prevention interventions developed or implemented by health promotion organisations or others working in prevention [6, 13, 36–38]. Evaluations relating to these projects are even more difficult to find [6, 13]. Content analyses of social networking sites showed that there are health promotion activities, such as information dissemination and social marketing campaigns, occurring on social media, but most were not documented in published literature [6, 13]. Gold et al. [13] found that health promotion activities on social media were most likely to be undertaken by not-for-profit organisations and government departments or agencies, with only 10% run by academic institutions. Approximately two-thirds of health promotion social networking sites existed to promote a service or organisation, while just under a third (29%) included a campaign or intervention [13]. A study from the United States found that 42% of community-based organisations had a social media presence, but they often had short reach, made limited use of the interactivity offered by the media, were unlikely to be updated and maintained over time, and were not evaluated or evaluation results were not disseminated [39].

Another part of this picture relates to publicly available smartphone applications (apps), which can be created by anyone, including health promotion agencies or departments. In 2016, on the two major mobile platforms (iOS and Android), more than 259,000 mHealth apps were available, up from 100,000 from the previous year [40]. mHealth apps (including those developed by health promotion agencies) have received criticism relating to lack of theoretical underpinnings, poor quality, promotion of unhealthy behaviours and, most importantly, little or no evidence of effectiveness for their intended purposes [36, 41–43]. A recent study found that there were no publicly available evaluations for any of the 29 apps created by Australian health promotion bodies [6].

In short, most technology-facilitated interventions produced in research do not end up benefiting non-research populations. Further, most of these interventions that are available outside of research settings are either undocumented or have little or no evidence of benefit [26]. Despite the problems in the current landscape, there are many advantages to these tools, and many reasons to work with them. The *Lancet* Commission on Adolescent Health and Wellbeing highlighted that digital media offer “*outstanding new possibilities for engagement*” with youth populations [44]. Brusse et al. [6] contended that health promotion practitioners have no option but to engage with technology, given its increasing ubiquity. Others have argued that health promoters need to engage with technology effectively in order to ‘fight fire with fire’, considering the successful and expanding uses of social media and smartphone apps by industries selling harmful products [4, 45–47].

It is important to acknowledge that the gap between research and practice is not unique to the technology-facilitated prevention field. There is a substantial body of evidence to show that the research and practice sectors tend to operate in silos, with barriers including different operating mechanisms, funding requirements, reward systems and priorities [27, 48–50]. However, we and others argue that given the rapid evolution of these technologies, and the large number of organisations and individuals involved as either producers or end-users of technology-facilitated prevention interventions, it is imperative that we specifically investigate translation in the technology field and explore opportunities to enhance collaboration between sectors [6]. We therefore aimed to explore the following research questions from the perspectives of health promotion experts and researchers working with technology-facilitated prevention:

1. What are the barriers and facilitators for health promotion practitioners in engaging with technology-facilitated prevention work?
2. How do researchers working with technology-facilitated prevention see their role in translation?
3. What are the barriers and facilitators for researchers in engaging with the translation of their technology-facilitated prevention work?

## Methods

### Study design

We utilised a realist qualitative study design [51], involving in-depth interviews with researchers working with mobile phone and social media interventions and prominent health promotion experts. A key purpose of the study was to inform the development of a quantitative survey of health promotion practitioners about use and evaluation of technology-facilitated prevention initiatives. We therefore sought to better understand the context in which practitioners operated, and gauge their perception of the state of play in the field. Due to the proliferation of research in technology-facilitated prevention, we decided that it was essential to also include researchers and explore their role in translation. Qualitative methods, particularly in-depth interviews, are a useful method for producing rich and detailed data in areas where less is known or documented [52]. The semi-structured nature of the interviews allowed flexibility for participants to move into discussions that were important to them, which may not have been captured in a structured interview or questionnaire [52].

### Sampling and recruitment

We selected participants purposively, based on their unique knowledge and insights into technology-facilitated prevention research and/or health promotion practice. This sampling technique involves the selection of experts based on their knowledge and experience of the core issues of the research [52]. We originally aimed to include participants from both Australian and international contexts; however, after piloting the interviews with two international experts, it became apparent that the prevention and translation contexts differed markedly from Australia. As such, we were concerned that we would be unlikely to reach data saturation or draw useful conclusions in this small, unfunded study. We subsequently only interviewed participants based in Australia going forward, and excluded the data collected from the two international participants for the analyses we report on in this paper.

For the sampling of researchers, a literature search was undertaken to identify prominent academics in the field who had been engaged with technology-facilitated prevention research for at least 3 years. We approached 12 researchers, 9 of whom consented to participate.

Health promotion experts were selected based on their senior leadership within the health promotion and prevention field in Australia, and were identified through their leadership positions within public health and health promotion associations and/or prominent publishing about health promotion and public health practice in textbooks or academic journals. Some health promotion experts also had experience in research, but were still considered to be health promotion experts if their work was mostly practice based. We consulted with 4 current senior health promotion practitioners and used snowball sampling during the course of the study to identify other health promotion experts. We approached 14 health promotion experts, and 9 consented to participate. Our final sample therefore consisted of 18 people.

Participants were approached via email, with a description of the study and were invited to respond if interested in participating. Depending on the participant’s location, interviews were conducted via telephone or in person. A semi-structured interview guide was developed by the researchers who consulted with other health promotion practitioners who were not involved in the study; this helped to inform language and terminology as well as topics and framing. Questions covered experience with technology-facilitated prevention, challenges and concerns related to relevant interventions, evaluation, dissemination of research/evaluations, perspectives on promising innovations with potential for translation, contact between health promotion practitioners and researchers about technology-facilitated prevention interventions, technological skills needed to work with mobile phones and social media for health promotion, and barriers and facilitators for translation of technology-facilitated prevention interventions. Interviews ranged from 45 to 80 min in length, and were audio-recorded and transcribed verbatim. The interviews were conducted by the primary author, who has qualifications and training in both technology-facilitated prevention research and health promotion practice. The researcher actively considered the potential for her position to influence the data collection and analysis process. As previously mentioned, she consulted extensively with health promotion practitioners prior to commencing the study to inform the interview guide and recruitment strategies. Another strategy used for reflexivity included writing field-notes prior to and following each interview in a reflexive journal to consider nuanced interactions which may not be well captured in audio-data. We also used peer debriefing following interviews as a strategy to facilitate reflexivity. To enhance rigour and reduce the chances of misinterpretation [53], the researcher conducting the interviews was careful to repeat statements back to the participants to clarify interpretations (known in qualitative research as ‘member checking’ [52]).

### Data analysis

We used an inductive approach to develop a coding framework. This involved reading the transcripts multiple times to first familiarise ourselves with the data, while making notes about potential codes to use to organise the data. These notes were used to develop a preliminary coding framework, which was refined iteratively during the process of coding the first four transcripts. Coding was then completed deductively using QSR NVivo V11 software by one researcher. A second researcher blind-coded a sample of four transcripts; the two researchers then met to check consistency and discussed discrepancies. Following coding, we searched for themes within the data; we used a realist or semantic approach, that is, the themes were identified based on explicit meanings of the data. At the time, we felt that this ‘data-driven’ approach was appropriate due to the pragmatic nature of the research questions and small scope of the study [51]. Following coding, we undertook a process of re-organising coding structures to consider relationships across codes in order to identify themes related to both researchers and practitioners. Themes were then reviewed and defined based on their importance to technology-facilitated prevention specifically.

## Results

The four key themes which provide insight into current issues affecting technology-facilitated prevention translation, namely the speed of technological developments, priorities for evidence, perceived roles in the translation continuum, and skills for translating and implementing technology-facilitated prevention, are described below.

### Speed of technological developments

The speed at which new technological developments emerged was described as a general challenge to working in technology-facilitated prevention, which also had strong implications for translation. Researchers described the importance of novelty when applying for grant funding and publishing work, and therefore felt immense pressure to keep pace. While this was seen to present exciting opportunities for innovation, researchers described obsolescence as a recurrent threat, and found it difficult to keep abreast of the current evidence, due to the large number of players in the field internationally. Researchers described that they found it increasingly challenging to work in the fast-paced research environment:

“*The last couple of years I’d say things have moved particularly fast. Obviously, that represents opportunities, but you also don’t know if things will have moved along too fast by the time you can publish.*”

(Researcher #7)

“*It’s definitely an exploding field. When I started a few years ago, I was one of the only people working in this particular area. There are literally now hundreds of papers each year coming out each year from every corner of the world. But it’s not just us. It’s doctors, allied health, computer science… It’s much harder now to say that you’re really doing something new and everyone’s comparing it to the kind of technology produced by Google wondering why you can’t just do what they’re doing. It’s getting harder*.”

(Researcher #2)

Researchers also described the conflict between this rapid pace and the slow and long process of building rigorous evidence for interventions, including various stages of research from formative research and pilot testing through to efficacy and effectiveness testing. This process was seen as necessary, but impeded their ability to produce relevant and timely evidence, which challenged the relevance of their work. A few researchers discussed that they had had to move on from a technology or before feeling satisfied that the evidence about its use was ‘ready’. One researcher felt strongly about the need for a more unified approach to evidence-building:

“*Everyone’s on apps now, but we really don’t even have cohesive evidence about text messaging because every study is so different. Everyone’s chipping away at different parts of the block and then we have to throw it out before it’s carved.*”

(Researcher #3)

“*Rigorous, ethical research takes these steps. But sometimes by the end of it, it turns out that that technology is on its way out. It’s a Catch-22.*”

(Researcher #1)

However, health promotion experts believed that the practice field needed to be ‘ready’ to accept the technology-facilitated prevention interventions developed in research in order for translation to take place. They reflected that the developments were sometimes out of sync with practice readiness, meaning that the health promotion agencies were often significantly lagging behind the commercial sector and research world in terms of their use of technology for health. One expert suggested greater involvement of practitioners in the design and selection of interventions in order to address this issue:

“*There’s a lot of conversations about knowledge translation from academia and I actually think there needs to be two-way conversation. I think that it’s really important for researchers to come out to the centre and say ‘Hey, this is what we’re thinking of testing’ and we’ll go ‘oh that’s very nice but it’s not gonna work’.*”

(Health promotion expert #1)

### Priorities for evidence

Both researchers and health promotion experts discussed study design and how the focus on controlled research settings, while necessary for securing competitive grants, could reduce the real-world applicability of the findings. One researcher described feeling conflicted because of this tension:

“*We have to do things a certain way to meet the expectations of the scientific community… But what we’re working on isn’t that easily replicated.*”

(Researcher #1)

“*It is always a tension… At the time that you’re designing* [a study] *you’re really just trying to get it right from a science perspective. There are so many different voices in the room and politics to balance. I’ve had it where you’ve got a professor on your team who’s an expert in behaviour change saying you need to do this this way, and you’re thinking ‘how do I fit in that kind of assessment into a brief survey?’. It just doesn’t really work on a mobile platform. And you’ve got excited young RAs* [research assistants] *saying you need to do this other thing because that’s how people are using the platform now. The old-school guy will usually get his way. Because you want to get it right.*”

(Researcher #9)

Similarly, a health promotion expert expressed some mistrust of intervention study findings due to questions of generalisability to community contexts.

“*You take out all of the white noise that is the real world. For mHealth, that could be the difference in whether anyone actually uses your app.*”

(Health promotion expert #9)

Concern about health equity and technology was a recurring subtheme with implications for translation. Several health promotion experts emphasised that their organisations were funded to reach marginalised populations, who tend to have lower access to technology. They described a sense of unease with current technology-facilitated prevention interventions, and concerns that they may only reach the healthiest and wealthiest within a population. Study design and research samples again emerged as a concern, with interviewees saying that they tend to engage “*low-hanging fruit*”, meaning mainstream populations. This de facto targeting led to health promotion experts questioning the relevance of study findings to the populations with which they worked. Despite these concerns, some health promotion experts discussed the growing use of technology across usual socioeconomic divides, and the opportunities technology provides for reaching populations that are traditionally difficult to access. However, these experts did not think that this potential was yet realised in either research or practice, as this quote demonstrates:

“*If technologies can be harnessed in the right way… There are brilliant opportunities for working with all of kinds of populations in ways that we can’t right now.*”

(Health promotion expert #6)

Some health promotion experts pointed out that most of the current technology-facilitated prevention research focused on behavioural interventions, which they regarded as only a small portion of their field’s work. For most experts, individual-level behaviour change was less important than health promotion activities focusing on addressing social determinants of health, particularly at the environment and policy levels. The following quotes from health promotion experts illustrate their views of the primary focus of their sector:

“*Our skill set is in the world of determinants-based health promotion. That’s what we do, we’re primary preventers.*”

(Health promotion expert #8)

“*We’ve been brought up on the Ottawa Charter – changing access, environments.*”

(Health promotion expert #2)

### Perceived roles in the translation continuum

Some researchers held the view that their role in translation was to produce and disseminate their research, so then it could be taken up by practitioners. One researcher described her understanding of the separate roles of researchers and practitioners:

“*We all have a role to play in translation. Research is about the production of knowledge. Practice is about implementing evidence-based programmes.*”

(Researcher #4)

However, insights from health promotion experts illustrate that linear knowledge translation is challenged by barriers to accessing evidence, including technical jargon, the omission of practical information such as intervention cost, and competing drivers for programme design, including political appeal and directives from management. One health promotion expert mentioned the financial barrier of access to journal articles, stating that many organisations could not afford subscriptions:

“*We had to rely on the fact that hopefully there’s someone on the team studying a Masters or something and we could use their login. Large health services have libraries, councils don’t. Small agencies… No.*”

(Health promotion expert #1)

Resources and time constraints emerged as major barriers to enacting roles of translation. Health promotion practitioners frequently referred to the poor investment in prevention in Australia, and how cuts in recent years had affected their organisation’s ability to meet community needs. One health promotion expert expressed the challenges of providing programmes in such a tight and unstable funding context:

“*We’re operating with nothing. Every year it’s less.*”

(Health promotion expert #3)

Insufficient funding was seen to directly affect health promoters’ ability to implement technology-facilitated prevention, which require considerable time and cost to develop. This point was mostly made with reference to mHealth rather than social media interventions. Three experts who had worked in health promotion management roles recounted stories of app development projects that cost significantly more than planned, and/or ended without a product being released. Conversely, social media interventions were seen as cheap; experts’ concerns related more to development skills and effective use of the technology, which will be discussed in the following section.

Health researchers similarly described their funding context as ‘limited’ and ‘challenging’. As mentioned above, they asserted that grant requirements largely dictated the nature of the work they produced in terms of study design and population samples. However, the funding context also more directly influenced their ability to engage with translation in partnership with external organisations. All but one researcher had received correspondence from practice and service organisations with interest in their technology-facilitated prevention work, but only one had ever engaged in a partnership to implement a technology-facilitated prevention strategy or intervention in a community setting. Health promotion experts currently working in practice frequently perceived that researchers were generally ‘too busy’ to engage with them. One health promotion practitioner described her experience of communicating with a researcher about his work:

“*You’ll contact them, and they’re nice, so interested, all of that but when it comes down to it, if you don’t have the funds then they’re not interested.*”

(Health promotion expert #4)

Some researchers gave insights into the other side of this picture, in terms of feeling genuinely overwhelmed and lacking the capacity to take on more work. One researcher lamented her inability to participate in translation-building activities such as communicating with practitioners, citing competing demands, job insecurity and poor work-life balance:

“*I’m working 6 days a week already… If I had time…*”

(Researcher #5)

Health promotion experts similarly portrayed practitioners as time poor and used to “*making do with what they’ve got*”. This sometimes necessitated shortcuts to ideal models of health promotion practice, and influenced practitioners’ use of evidence and formative, process and impact evaluations. Experts agreed that funding was therefore an important practical barrier to translation for the emerging area of technology-facilitated prevention. Partnerships were viewed as a strategic way to pool knowledge and enhance evaluation capacity. However, health promotion experts described wariness about engaging researchers due to the delays that applying for ethics approval often involved, taking into consideration the short-term funding normally granted for health promotion projects. One expert complained that ethics committees were ‘out of touch’ with what community work looked like and created lengthy delays in project implementation as a result. This was a key barrier to translational partnerships, which were seen as essential, if difficult, for improving translation of technology-facilitated prevention interventions from research to practice. One health promotion expert identified the funding contexts of each sector as key barriers to partnerships:

“*Clearly we have to work together more. But considering their funding and our funding and the way we work separately – I don’t know.*”

(Health promotion expert #1)

### Skills for translating technology-facilitated prevention

Health promotion experts described their workforce as generally having basic proficiencies with technology. While the technology skill level was seen as adequate to undertake the core components of a practitioner’s role, health promotion experts speculated that it made engaging with technology-facilitated prevention intimidating and practically difficult. One health promotion expert described it as being a knowledge and skill requirement that added to already over-loaded expectations:

“*For some reason we have an expectation that a health promoter needs to be a planner, an evaluator, and a graphic designer and health literacy specialist, a health communication specialist… They’re supposed to do this in a 3-year degree when they’re taught none of those skills.*”

(Health promotion expert #1)

Similarly, researchers did not see themselves as having high levels of skill in relation to technology; they saw their main expertise to be with a particular health topic or behaviour such as diet or sexual health. One researcher mentioned that she often encountered assumptions that she was a general technology expert, but in reality had a much smaller niche area of knowledge:

“*You work within a particular health area and that’s where your knowledge is. You just happen to work with SMS etc. I don’t know anything about social media, for instance.*”

(Researcher #6)

Researchers explained that they would usually contract out highly technical aspects of their work to others such as software developers. Subcontracting of consultants with technical skills was deemed necessary for most projects, and was built into budgets during grant applications. One researcher plainly stated that a high level of technological skill was not required to undertake technology-facilitated prevention research:

“*I’m not a programmer. We get programmers in for that.*”

(Researcher #1)

Health promotion experts believed that practitioners lacked access to sufficient funding to outsource technical components, and therefore had no choice but to do the work themselves. One health promotion expert with significant experience in management explained that they did their best with whatever funding was available:

“*It comes down to budget… When the staff in my team are going to develop an app I’m going to get them to watch some videos about how to write an app and do some reading online because we do not have the funding to engage a creative agency that’ll do that stuff for us.*”

(Health promotion expert #9)

This dilemma about skills and funding was seen to directly affect the capacity of health promotion practitioners to engage with technology-facilitated prevention interventions. Health promotion experts described a greater level of comfort about use of social media than mobile phones (for intervention purposes). However, some questioned whether social media was currently being used effectively within health promotion organisations:

“*Every org*[anisation] *has their own Facebook page. But what for? How often is it updated, do people actually use it? I don’t think we’ve got a good idea of careful, purposeful use. We just know that we are supposed to have them and it’s now pretty easy to do. Every boss wants to do an app. But they don’t know what it’s for.*”

(Health promotion expert #5)

Researchers also focused on specific skills for translation, with several pointing out that they and other researchers might not know how to engage in the translation process, even when they were interested in it. One researcher said that it was outside of the scope of his department’s focus, capacity and skillset:

“[Translation] *is not part of the system, currently at least. I don’t know what it would even look like, what the tasks would be.*”

(Researcher #9)

## Discussion

Our findings suggest that there are significant barriers to translation of technology-facilitated prevention interventions. Some findings mirror those of other studies of health promotion practice, which found that competing priorities, resource limitations and organisational capacity are important in determining use of evidence in programme planning, engagement in translation and evaluation practice [49, 54–56]. We add to this literature by highlighting barriers more specifically related to technology-facilitated prevention, such as the pace of technological development, and how these clash with the time taken to develop and ready evidence for translation.

Previous studies in the wider prevention sector have found, as we did, that the differing value systems and priorities of research, practice and policy can be barriers to translation of health promotion interventions [27, 57, 58]. In our study, researchers and health promotion practitioners saw their roles as distinctly separate, and found it difficult to envisage greater collaboration across sectors in their current working context.

Our finding regarding health promotion practitioners’ concerns about equity in technology-facilitated prevention interventions is an important contribution to the discussion, as it highlights a key contention surrounding the populations targeted by each sector. Further discussion of the practical relevance and genuine scalability of the interventions currently being produced in the research field is warranted [26]. Studies have often shown that there is a digital divide, with those of low socioeconomic status, non-English speaking background, older age and lower health literacy being less likely to engage with health-related content on mobile and internet platforms [59–61]. This divide has important implications for researchers in terms of the technologies that they test and the populations with which they test it.

Although mobile phone access is more ubiquitous across socioeconomic spectrums than internet access [62], recent research shows that there are still underserved sub-groups such as those with lower educational attainment [60]. Baum et al. [63] contend that a ‘vicious cycle’ occurs when information technology access and literacy is assumed in health promotion activities. They argued that the increasing focus on digital delivery of health information and interventions further excludes the marginalised, and urged health promoters to continue to offer strategies in traditional forms [63]. Though it is likely that technology literacy and use will increase as availability and affordability improve, it is important to consider that newer, more sophisticated technologies will continue to emerge and will not be affordable to all. It is understandable that researchers will want to harness newer technologies and understand their potential uses for improving health, but consideration of access is integral to scalability, relevance and future translation. Future research and evaluations of the use of technology-facilitated prevention interventions in vulnerable populations would also be valuable [6].

Researchers in our study were predominantly used to a linear, one-way form of translation, with their primary role in the sequence being the simple dissemination of research; this finding is consistent with previous research [57, 64]. The researchers’ description of translation most closely resembled Rychetnik’s model of translation processes to support evidence-based policy and practice [65]. In this model, there are five key stages, namely (1) problem definition, (2) solution generation, (3) intervention testing, (4) intervention replication and (5) intervention dissemination. However, this type of linear process (sometimes termed as the ‘pipeline fallacy’ [66]) has been criticised as slow and ineffective for producing real-world benefits [35, 67, 68]. Updates to Translation Continuum (‘T’) models demonstrate a more complex route to achieving real world impact [69]. The T0–T4 model includes five stages, from problem definition (T0) to discoveries (T1), tests of interventions (T2), the production of evidence-based recommendations (T3), to the implementation of interventions into organisations and communities (T4). A key feature of this model is that, between each of these stages are bi-directional arrows, and at each stage, stakeholder engagement and evidence integration occurs. The overall process is also seen to be cyclical, with evidence from programme implementation then informing problem definition. The researchers in our study were predominantly focused on the T1 and T2 stages of translation, with limited engagement or discussion of the other stages. We recommend that researchers consider their work in the context of the larger translational machine, and that they include stakeholder engagement with practitioners and community at the various stages of intervention research.

Researchers could also consider the use of study designs that allow for the simultaneous study of both effectiveness and implementation. The hybrid effectiveness–implementation models described by Wolfenden et al. [64] are promising in terms of both reducing the time taken for research to be produced and disseminated, and for increasing the relevance of findings for real-world populations. Milat et al. [27] have previously advocated for a co-production model in which practitioners and researchers are involved from inception to dissemination of research. This model may address many of the barriers to translation raised in our study by pooling skills, resources and knowledge, and enhancing the real-world relevance of the interventions produced. Although there are likely challenges in merging the operations and priorities of research and practice sectors, there is evidence to suggest that research produced under this model is more relevant to end-users and more likely to be integrated into policy and practice [70].

While research and practice fields continue to operate separately under their respective funding streams, it is hard to see how translation will improve. Our findings support the idea that the relevance of technology-facilitated prevention research could be enhanced through greater consultation with end users such as health promotion practitioners, at the very least. We concur with previous recommendations that researchers should commit to disseminating their work in widely available sources (such as open-access journals) and with transparent accounting of the time, resources and expertise needed to implement their interventions [29–31]. However, it is clear that there needs to be greater incentive for researchers and practitioners to work together, given the resource constraints and perceptions of both technology and research–practice partnerships in each field. Therefore, it is vital to consider improvements to funding schemes in both research and practice to support translation.

Further funding should be invested in translational research that includes research–practice partnerships. Some investments have been made in Australia, but these currently account for a small proportion of research funding. In 2016, approximately 3% of the Australian National Health and Medical Research Council’s competitive grant funding went towards translation-specific funding through the Partnership Grants scheme, the Translation of Research Into Practice Fellowships and the Translational Research Projects for Improved Health Care scheme [71]. A further issue with the Partnership Grant scheme is the requirement of matched co-funding from partner organisations; this scheme is derived from a traditional model of significant long-term profit opportunities for partners, which may never eventuate for health promotion organisations, meaning that these grants are out of reach.

Challenges to using evidence in health promotion practice and policy have been described, as practitioners and managers seek evidence that is relevant to their context and population, has high external validity, and will meet the needs of managers and funding bodies [54, 72]. Practitioners may know what high-quality evidence is; however, structural demands influence what they can use in practice. In addition to ongoing emphasis on evidence-based practice, it is important to address these organisational, funding and policy levers from both ends in order to overcome these barriers to translation.

For health promotion practice, increased funding is also required to enable greater investment in formative research, pilot programmes and evaluation. Further documentation and evaluation of technology-facilitated prevention work is essential for building the evidence base for what does work in real-world practice, and with the populations and strategy types prioritised by the health promotion sector. More information on development, implementation and evaluation from these projects would be useful to guide future work, and to ensure that practitioners have a stronger voice in the evolution of this field. The co-production model described above could be beneficial for ensuring that projects are adequately documented and disseminated in places where both researchers and practitioners will find it. Where co-production does not occur, health promotion funding schemes need to allow for evaluation in their budgets, whether this be for research–practice partnerships for evaluation, or to allow practitioners the time and resourcing to undertake it in-house. Contract timeframes should allow flexibility for ethics approval lead times and sufficient evaluation activities at the completion of projects. Efforts have been made to provide specific guidance for evaluating technology-facilitated prevention interventions [38, 73].

## Limitations

We selected researchers and health promotion experts who were appropriate for our research objectives, but in this complex and rapidly-changing field, it is possible that our study was not able to capture the full range of perspectives on this topic. Our study was limited in focusing on the Australian context. Further, the primary researcher collecting the data for the study was susceptible to interviewer bias due to her experience in technology-facilitated prevention research. However, the researcher reflected upon this bias critically throughout the study, and benefited from the input of experienced health promotion practitioners and researchers during the analysis process.

## Conclusion

In order to maximise the vast potential of technology-facilitated prevention interventions to promote population health, it is essential that translation is at the forefront for both researchers and practitioners. Both researchers and practitioners can take action to improve translation of technology-facilitated prevention interventions. For researchers, this includes designing interventions with real-world populations and contexts in mind, consulting with practitioners, and committing to widespread, transparent dissemination of their work. For practitioners, more work is needed to document and evaluate technology-facilitated prevention interventions implemented in community settings. However, enhanced knowledge translation and collaboration is unlikely to improve without changes to funding schemes to incentivise and enable research–practice partnerships.

## Abbreviations

eHealth: electronic health
mHealth: mobile health
